# Cellular and Molecular Mechanisms of Oxidative DNA Damage and Repair

**DOI:** 10.3390/medicina61112013

**Published:** 2025-11-11

**Authors:** Adnan Ayna, Cuneyt Caglayan, Seyithan Taysi

**Affiliations:** 1 Department of Chemistry, Faculty of Science and Literature, Bingol University, 12000 Bingol, Turkey; aayna@bingol.edu.tr; 2 Department of Medical Biochemistry, Faculty of Medicine, Bilecik Seyh Edebali University, 11230 Bilecik, Turkey; cuneyt.caglayan@bilecik.edu.tr; 3 Department of Medical Biochemistry, Faculty of Medicine, Gaziantep University, 27410 Gaziantep, Turkey

**Keywords:** cell damage, DNA damage, DNA repair mechanisms, free radicals, stresses

## Abstract

DNA is continuously exposed to endogenous and exogenous factors that induce oxidative modifications leading to mutations and genomic instability. Oxidative DNA damage plays a dual role, contributing to physiological signaling at low levels while promoting mutagenesis, carcinogenesis and degenerative diseases when unpaired. Among various lesions, an oxidized base, such as 8-oxo-2′-deoxyguanosine (8-oxodG), is one of the major biomarkers of oxidative stress and genomic damage. Cells have evolved sophisticated repair processes, including base excision repair (BER), nucleotide excision repair (NER), and mismatch repair (MMR), to maintain genomic integrity. Dysregulation or polymorphism of these repair genes has been linked with cancer, neurologic, and cardiovascular disorders. This review discusses an overview of what is presently known concerning oxidative DNA damage and repair mechanisms, particularly emphasizing their molecular players, signaling routes, and human disease implications. It further refers to the latest advances in CRISPR-based technologies and multi-omics approaches that are redefining our understanding of DNA damage response (DDR) networks and creating new frontiers for therapeutic interventions.

## 1. Introduction

The cell, the fundamental unit of living organisms, possesses a highly intricate network of biochemical pathways collectively referred to as the DNA damage response (DDR). This system functions to preserve genome integrity by preventing the transmission of deleterious mutations to subsequent generations. Despite the inherent stability of DNA as an organic macromolecule, the genome remains continuously exposed to various endogenous and exogenous sources of damage [1].

In living organisms, the oxygen absorbed through respiration is partially converted into free radicals as a byproduct of normal metabolic processes. Free radicals, characterized by the presence of unpaired electrons, are highly reactive molecular species [2,3]. Cells possess an elaborate oxidant/antioxidant defense system designed to detoxify these reactive molecules. When reactive oxygen species (ROS) [4], reactive nitrogen species (RNS) [5], and other radicals are produced excessively and exceed the cell’s detoxification capacity, oxidative, nitrosative, and related types of stress arise. These stress conditions can inflict significant damage to the cellular components. Playing an important role in living organisms and as a genetic material, DNA must be preserved to ensure the faithful transmission of hereditary material. However, it remains vulnerable to endogenous and exogenous sources of damage [6,7].

Oxidative DNA refers to the structural and chemical alterations in DNA induced by ROS, which are oxygen-containing and highly reactive molecules. These reactive species can interact directly with DNA, resulting in single-strand breaks (SSB), double-strand breaks (DSB) and various base modifications [8,9]. Such damage is recognized as one of the major contributors to aging and the onset of certain diseases [10], particularly cancer making it critical area of biomedical research. Oxidative DNA lesions can introduce mutations within genes, potentially leading to malignant transformation of genetic defects associated with hereditary disorders [11]. To mitigate these detrimental effects, cells have evolved multiple repair mechanisms, including base excision repair (BER), nucleotide excision repair (NER), and homologous recombination (HR). These repair processes function to identify, excise and replace the damaged DNA bases, thereby restoring the integrity of the DNA molecule [12]. By studying oxidative DNA damage, scientists can better understand the mechanisms that cause these health problems and develop treatments and preventive measures to reduce their occurrence. The major objective of this review is to provide a unified overview of the molecular and cellular mechanisms underlying oxidative DNA damage and repair. It specifically discussed the main oxidative DNA base modifications and their biological consequences, described the key enzymatic mechanisms employed in their detection and repair, and discussed the pathophysiological consequences of defective repair processes. It also highlighted contemporary technological advances such as CRISPR-mediated genome editing and omics approaches that are changing our understanding of DDR and enabling new diagnostic and treatment possibilities. Overall, this review seeks to integrate current understanding of oxidative DNA damage with new advances in repair control and translational significance and offer a framework for future investigation that integrates molecular mechanisms with disease pathology and the development of targeted therapy.

## 2. The Molecular Mechanisms of DNA Damage

Oxidative damage to DNA bases can occur via various mechanisms. One of the most common is the formation of ROS such as hydroxyl radicals (^•^OH), superoxide anions (O_2_^•−^), and hydrogen peroxide (H_2_O_2_). These ROS can interact with the DNA bases, resulting in the creation of modified bases such as 8-oxo-7,8-dihydro-2′-deoxyguanosine (8-oxo-dG) [13]. This modified base can then lead to mutations in the DNA sequence. Another mechanism of oxidative damage to DNA bases is through the formation of lipid peroxidation products [14]. Lipid peroxidation occurs when the double bonds in unsaturated fatty acids are broken down by ROS, resulting in the creation of various reactive aldehydes and other compounds [15]. These can then react with the DNA bases, leading to mutations. Finally, oxidative damage to DNA bases can also occur through the direct action of ROS on the DNA backbone. This can lead to the occurrence of abasic sites, which may then lead to mutations in the DNA sequence [16].

### 2.1. Oxidation of Guanine to 8-Oxo-7,8-dihydroguanine (8-OxoG) and 8-Oxo-dG

**Figure 1:**
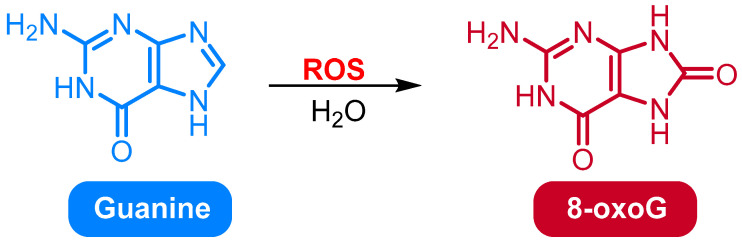
Oxidation of guanine to 8-oxo-7,8-dihydroguanine (8-oxoG).

Among numerous oxidative DNA damage products, 8-oxoG and its deoxynucleoside derivative 8-oxodG are the most widespread and well-studied biomarkers of oxidative stress. 8-oxoG is formed via the oxidation of the guanine base at the C8 position to 8-oxo-7,8-dihydroguanine (Figure 1), a form of oxidized nucleobase that can exist in free or nucleic acid-bound form [17]. When this oxidized base is added to a deoxyribose sugar, 8-oxodG, the most frequent oxidative DNA lesion, is generated [18]. 8-oxodG is also widely accepted as a stable biomarker of oxidative DNA damage in cells and body fluids such as urine, plasma, and serum [19]. It is typically formed by the attack of ROS, particularly hydroxyl radicals (^•^OH), on the guanine base due to its lowest redox potential among DNA bases [17]. Functionally, 8-oxoG or 8-oxodG in DNA is capable of inducing mutagenic base mispairing because 8-oxoG prefers to pair with adenine instead of cytosine when DNA is replicated, leading to GC → TA transversion mutations [20]. Since its recognition by repair enzymes and its mutagenic activity both depend on subtle structural and conformational differences in the double helix structure of DNA, knowledge about its molecular geometry is critical to knowing how it causes base mispairing and how repair mechanisms such as OGG1 recognize and remove it. To generate such structural data, advanced spectroscopic techniques have been utilized. Among these, nuclear magnetic resonance (NMR) spectroscopy is an effective method to probe the three-dimensional structure and dynamics of 8-oxoG in DNA. NMR spectroscopy enables the measurement of chemical shifts and coupling constants of nuclei in 8-oxoG, providing details into its electronic environment, hydrogen bonding, and base pairing conformations. This information helps determine how 8-oxoG alters the localized DNA architecture, and therefore its engagement with polymerases and repair enzymes [19,20].

#### 2.1.1. Clinical Implications of 8-oxoG and 8-oxodG and Their Diagnostic and Prognostic Potential

Oxidative nucleic acid biomarkers, particularly 8-oxodG and 8-oxoG, have central roles in the pathogenesis and development of cancers, neurodegenerative diseases, diabetes mellitus, and cardiovascular disease [18]. In colorectal cancer, increased plasma and urinary 8-oxodG levels correlate with tumor development and metastasis, confirming their potential for early detection [21]. Similarly, in gastric cancer, *Helicobacter pylori*-induced oxidative stress augments 8-oxodG accumulation, and downregulation of the DNA repair enzyme (HOGG1) increases damage further [22]. Higher levels of 8-oxodG in chronic atrophic gastritis and gastric cancer suggest its predictive value. In breast cancer, tissue and urinary 8-oxodG are elevated preoperatively but return to normal upon tumor removal, suggesting tumorigenesis dynamics of oxidative stress; low tissue 8-oxodG, on the other hand, correlates with aggressive phenotypes, suggesting complex oxidative pathways [23]. High biomarkers of oxidative damage also characterize lung and ovarian cancer and correlate with elevated 8-oxodG, which is predictive of poor survival [24,25]. In neurodegenerative diseases, abnormally high oxygen demand and limited antioxidant capacity make neurons susceptible to ROS-induced nucleic acid damage. 8-oxoG accumulation in brain tissue and cerebrospinal fluid is evident in Alzheimer’s and Parkinson’s disease, and disease severity and progression are correlated [26]. Oxidized RNA and damage to mitochondrial DNA in early Alzheimer’s indicate oxidative stress as a significant pathogenic factor, while in Parkinson’s, increased 8-oxoG levels in CSF and serum, especially in early cases, suggest diagnostic value. Similarly, epilepsy and other neurological conditions such as dementia with Lewy bodies, prion disorders, and ALS show high 8-oxodG/8-oxoG expression, again highlighting the involvement of oxidative RNA and DNA damage in neuronal degeneration [27,28]. In diabetes mellitus, serum 8-oxodG level correlates with prediabetes and type 2 diabetes, correlated with body mass index (BMI) [29]. All of these collectively highlight the fact that oxidative nucleic acid biomarkers are not just markers of disease state but also therapeutic and preventive targets. Antioxidants such as fisetin, coenzyme Q10, and polyphenols of dietary origin have been shown to provide protective effects against ROS-induced damage, but high-dose supplementation can be harmful [30]. Monitoring 8-oxodG/8-oxoG changes is a measure of treatment effectiveness that can be quantified, especially for oxidative stress disease. Of the analytical methods listed, ELISA, GC-MS, CE-ECD, and HPLC-MS/MS have improved biomarker detection with increased sensitivity and specificity [31,32]. Of these, HPLC-MS/MS is still the most precise due to its specificity and reproducibility. Further advancement of these analytical platforms and mechanistic insights into oxidative stress will further support the clinical utility of 8-oxodG and 8-oxoG as diagnostic, prognostic, and therapeutic biomarkers for a wide range of oxidative stress-related diseases.

#### 2.1.2. Challenges and Limitations in the Clinical Application of 8-oxoG as a Biomarker of Oxidative DNA Damage

The ability of 8-oxoG to act as an oxidative DNA damage biomarker has long been recognized. Clinical use is prohibited by several issues yet to be clarified. One of them is the variance in study results. Certain research has reported elevated levels of 8-oxoG in various cancers, while others have not reported any noteworthy correlation with disease presence or development [33]. These differences owe their origins to differences in sample types, detection methods, and patient populations, and, as such, a need for protocols to be standardized. Secondly, 8-oxoG’s specificity as a biomarker is debated. Unlike 8-oxodG, 8-oxoG may also derive from RNA or the nucleotide pool, hence the susceptibility to misinterpretations when examining DNA damage [34]. This leads one to question the accuracy of 8-oxoG measurements in clinical trials. Another limitation is that there is no agreement on the best detection methods. Although high-performance liquid chromatography with electrochemical detection (HPLC–ECD) is widely employed, problems like overestimation caused by artifact oxidation at DNA isolation and hydrolysis steps have been noted [35]. These methodological drawbacks highlight the need for stable and reproducible analytical methods. Moreover, the clinical efficacy of 8-oxoG is compromised by its inability to quantify the extent of oxidative DNA damage. It has been shown that 8-oxoG levels may not be able to correlate with disease severity or therapeutic response and warrant exploring alternative biomarkers or combination approaches toward enhanced diagnostic and predictive accuracy. While 8-oxoG is a potential biomarker for oxidative DNA damage, it is undermined in clinical utility by issues in study variability, specificity, detection modes, and clinical significance [34]. Resolution of these issues through standardized protocols, improved detection methods, and full clinical studies is essential to its translation as an acceptable biomarker for disease diagnosis and prognosis.

### 2.2. Oxidation of Adenine to 8-Oxo-7,8-dihydroadenine

**Figure 2:**
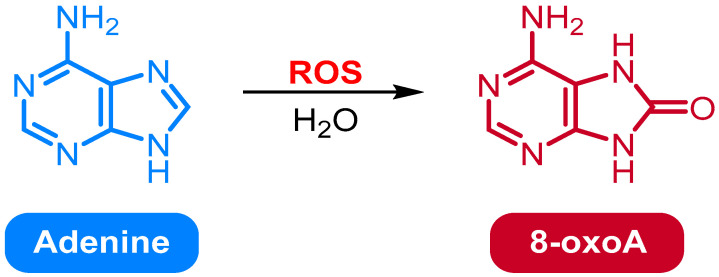
Oxidation of adenine to 8-oxo-7,8-dihydroadenine (8-oxoA).

Adenine can be oxidized to 8-oxo-7,8-Dihydroadenine (8-oxoA) by a variety of oxidizing agents, such as H_2_O_2_, ozone, and peroxynitrite (Figure 2). The reaction is catalyzed by enzymes, such as xanthine oxidase (XO), myeloperoxidase, and NADPH oxidase. The reaction proceeds via the formation of an intermediate radical, which is then converted to the final product. The reaction is reversible, and the product can be reduced back to adenine [36].

The oxidation of adenine to 8-oxoA is an important reaction in the metabolism of nucleic acids and has implications for the regulation of gene expression. 8-oxoA is an unstable compound that can cause mutations in DNA and RNA, which can lead to diseases such as cancer. Therefore, it is crucial to comprehend the mechanism of this reaction and to develop strategies to control it. Adenine can be oxidized to 8-oxoA by H_2_O_2_ and iron (III) in aqueous solution. The reaction is catalyzed by iron (III) and proceeds in two steps. In the first step, H_2_O_2_ oxidizes adenine to 8-hydroxyadenine. In the second step, 8-hydroxyadenine is further oxidized to 8-oxoA [37,38,39]. The overall reaction can be written as follows:

Adenine + H_2_O_2_ + Fe^3+^ → 8-oxoadenine + H_2_O

The reaction is catalyzed by iron (III), which acts as an electron acceptor. Iron (III) is reduced to iron (II), which is then oxidized back to iron (III) by H_2_O_2_. This cycle of oxidation and reduction allows the reaction to proceed.

### 2.3. Oxidation of Cytosine to 5-Hydroxycytosine 

The oxidation of cytosine to 5-hydroxycytosine (5-hC) is a reaction catalyzed by the enzyme cytosine deaminase. In this reaction, the enzyme removes an amino group from cytosine, resulting in the formation of 5-hC. The reaction is reversible, and the reverse reaction is catalyzed by 5-hydroxycytosine reductase. This reaction is important in the regulation of gene expression, as 5-hC is a modified form of cytosine that can be recognized by certain proteins [40].

#### 2.3.1. Oxidation of Cytosine to 5-hC in DNA by the Alkb Family of Enzymes

The AlkB family of enzymes consists of a group of dioxygenases that are responsible for the oxidation of cytosine to 5-hC in DNA. This oxidation is an important step in the regulation of gene expression and is essential for the proper functioning of the cell. The AlkB family of enzymes uses a two-step process to oxidize cytosine to 5-hC [41]. In the first step, the enzyme uses a dioxygenase reaction to oxidize the cytosine to form an intermediate product, 5-carboxycytosine (5-caC). In the second step, the 5-caC is converted to 5-hC by a hydroxylation reaction. The AlkB family of enzymes is found in both prokaryotic and eukaryotic cells, being involved in a variety of biological processes, including DNA repair, epigenetic regulation, and transcriptional regulation [42,43,44].

#### 2.3.2. The Role of 5-hC in DNA Methylation and Epigenetic Regulation

5-hC may act as an epigenetic modifier of the DNA base cytosine that is involved in a variety of important biological processes, including DNA methylation and epigenetic regulation [44]. DNA methylation is a process by which methyl groups are added to the DNA molecule, altering gene expression without changing the underlying DNA sequence. 5-hC is an important component of this process, as it is the primary target for DNA methyltransferases, which catalyze the transfer of methyl groups to DNA [45].

5-hC is an important intermediate in the repair of oxidative DNA damage, which is caused by ROS. Oxidative damage to DNA can lead to mutations, which can lead to cancer and other diseases. 5-hC is involved in the repair of oxidative damage by acting as a substrate for the base excision repair (BER) pathway. In this pathway, 5-hC is converted to 5-hydroxymethylcytosine by the enzyme thymine DNA glycosylase. 5-hydroxymethylcytosine is then converted to cytosine by the enzyme ten-eleven translocation enzymes. This process helps to restore the original cytosine base in the DNA, preventing the accumulation of mutations. 5-hC is also involved in the repair of oxidative damage to mitochondrial DNA, which is important for maintaining mitochondrial function. Thus, 5-hC plays an important role in the repair of oxidative DNA damage, which is essential for maintaining genomic integrity and preventing diseases [46,47].

### 2.4. Oxidation of Thymine to 5-Hydroxyuracil (5-hU)

Oxidation of thymine to 5-hydroxyuracil (5-hU) is a reaction that occurs in DNA when it is exposed to ROS. The reaction is catalyzed by an enzyme called thymine glycolase, which oxidizes the methyl group of thymine to a formyl group. The formyl group is then oxidized to a hydroxyl group, resulting in the formation of 5-hU [48,49].

#### 2.4.1. The Role of Oxidative Stress in the Oxidation of Thymine to 5-hU

Oxidative stress is a major factor in the oxidation of thymine to 5-hU. Oxidative stress occurs when there is an imbalance between the production of ROS and the ability of the cell to neutralize them. ROS are highly reactive molecules that can damage DNA, proteins, and lipids. When there is an excess of ROS, they can react with thymine, leading to the formation of 5-hU [48]. This is a common form of DNA damage that can lead to mutations and other genetic abnormalities. Oxidative stress can be caused by a variety of factors, including environmental pollutants, radiation, and certain drugs. It can also be caused by an imbalance in the body’s antioxidant defense system, which is responsible for neutralizing ROS. When the antioxidant defense system is not functioning properly, ROS can accumulate and damage DNA [50]. The oxidation of thymine to 5-hU is a major source of DNA damage, and it can lead to mutations and other genetic abnormalities. Therefore, it is important to reduce oxidative stress and maintain a healthy antioxidant defense system in order to reduce the risk of DNA damage.

#### 2.4.2. The Role of Iron in the Oxidation of Thymine to 5-hU

Iron is an essential element in the oxidation of thymine to 5-hU and a cofactor in the enzyme thymine dioxygenase, which catalyzes the oxidation of thymine to 5-hU. The enzyme uses two molecules of oxygen to convert thymine to 5-hU, and the iron atom is essential in the binding of the oxygen molecules to the enzyme. Iron also helps to stabilize the enzyme during the oxidation reaction. Without iron, the enzyme would not be able to catalyze the oxidation of thymine to 5-hU [51,52].

#### 2.4.3. The Kinetics and Mechanism of the Oxidation of Thymine to 5-hU

The oxidation of thymine to 5-hU is a two-step process that involves the formation of a thymine radical and the subsequent oxidation of the radical to form 5-hU. The reaction is catalyzed by a variety of oxidizing agents, such as H_2_O_2_, ozone, and oxygen. The first step of the reaction involves the formation of a thymine radical. This is achieved through the abstraction of a hydrogen atom from the thymine molecule by the oxidizing agent. The resulting radical is highly reactive and can react with oxygen to form a peroxyl radical. The second step of the reaction involves the oxidation of the thymine radical to form 5-hU. This is achieved through the addition of a hydroxyl group to the thymine radical. The resulting product is 5-hU, which is a stable molecule [53,54].

The overall reaction can be represented as follows:

Thymine + Oxidizing Agent → Thymine Radical + Hydrogen Atom

Thymine Radical + Oxygen → Peroxyl Radical

Peroxyl Radical + Hydroxyl Group → 5-Hydroxyuracil

The kinetics of the reaction are affected by the nature of the oxidizing agent and the concentration of the reactants. Generally, higher concentrations of the reactants and stronger oxidizing agents result in faster reaction rates. The reaction is also affected by the pH of the solution, with higher pH values resulting in faster reaction rates.

### 2.5. Deamination of Cytosine to Uracil

**Figure 3:**
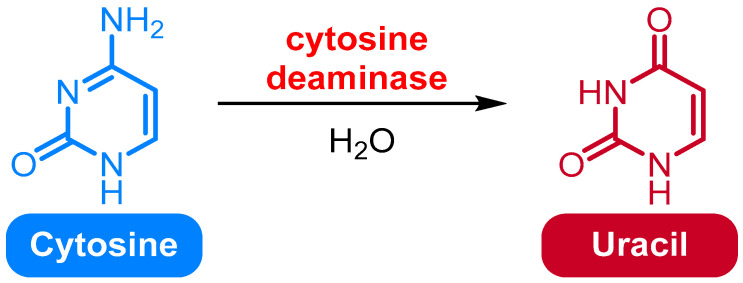
Deamination of cytosine to uracil.

Cytosine deamination is a chemical reaction in which the cytosine base in DNA is converted to uracil (Figure 3). This reaction is catalyzed by deaminases, enzymes that remove an amino group from the cytosine base. The reaction is irreversible, and the uracil is not replaced by cytosine. This reaction can lead to mutations in the DNA sequence, and thus is not incorporated into the newly synthesized DNA strand [40].

Apolipoprotein B mRNA Editing Enzyme, Catalytic Polypeptide-Like 3G (APOBEC3G) is a member of the APOBEC family of proteins, which are involved in the deamination of cytosine to uracil in human cells [55]. This process is known as cytidine deamination and is essential for the editing of mRNA transcripts. APOBEC3G is a single-stranded DNA-binding protein that is expressed in many cell types, including T cells, B cells, and macrophages [56]. It is involved in the editing of both coding and non-coding regions of mRNA transcripts, and it is thought to be involved in the regulation of gene expression.

### 2.6. Deamination of Adenine to Hypoxanthine

**Figure 4:**
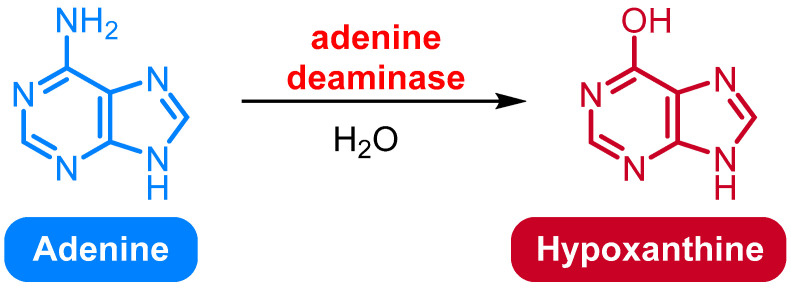
Deamination of adenine to hypoxanthine.

Adenine can be deaminated to hypoxanthine through the action of the enzyme adenine deaminase (ADA) (Figure 4). ADA is an enzyme that catalyzes the conversion of adenine to hypoxanthine. The reaction is an important part of purine metabolism and is essential for the maintenance of the cellular adenine nucleotide pool [57].

The reaction is also affected by temperature, with an increase in temperature resulting in an increase in the rate of the reaction. The reaction is also affected by the presence of inhibitors, such as allopurinol, which can inhibit the activity of hADA [58,59].

### 2.7. Formation of DNA–Protein Crosslinks

DNA–protein crosslinks are formed when a covalent bond is formed between a DNA base and an amino acid residue on a protein. This can occur through a variety of mechanisms, such as through the formation of a Schiff base between the DNA base and the amino acid residue, or through the formation of a disulfide bond between two cysteine residues on the protein. DNA–protein crosslinks can also be formed through the action of reactive oxygen species, which can cause oxidation of the DNA base and the amino acid residue [60,61].

### 2.8. Formation of DNA–DNA Crosslinks

DNA–DNA crosslinks are formed when two strands of DNA become covalently linked together [62]. This can occur through a variety of mechanisms, including the action of ROS, ultraviolet light, or certain chemical agents. Crosslinks can also be formed through the action of enzymes, such as topoisomerases, which are involved in the regulation of DNA topology [63]. Crosslinks can also be formed through the action of DNA repair enzymes, which can recognize and repair damaged DNA strands.

### 2.9. Formation of DNA–RNA Crosslinks

DNA–RNA crosslinks can be formed through a variety of mechanisms. One way is through the covalent attachment of a DNA base, such as adenine, to a ribonucleotide, such as uracil. This can occur through the formation of a Schiff base, which is a type of covalent bond between an aldehyde or ketone group and a primary amine. Another way is through the formation of a phosphodiester bond between the 5′ phosphate of a DNA nucleotide and the 3′ hydroxyl of a ribonucleotide. This type of bond is formed through a condensation reaction, which involves the removal of a water molecule [64].

### 2.10. Formation of DNA Single-Strand Breaks

DNA single-strand breaks (SSBs) can be formed in a variety of ways, including exposure to ionizing radiation, exposure to certain chemicals, and enzymatic cleavage. Ionizing radiation can cause SSBs through direct damage to the DNA molecule, while exposure to certain chemicals can cause SSBs by breaking the hydrogen bonds between the two strands of the DNA molecule. Enzymatic cleavage can also cause SSBs, as certain enzymes are capable of cleaving the phosphodiester bonds between the nucleotides of the DNA molecule [65,66,67].

### 2.11. Formation of DNA Double-Strand Breaks

DNA double-strand breaks (DSBs) are formed when both strands of the DNA molecule are broken. DSBs can be caused by a variety of environmental factors, such as radiation, chemicals, and mechanical stress [68,69]. They can also be caused by enzymatic activity, such as endonucleases, which are enzymes that cleave the phosphodiester bonds of DNA [70]. DSBs can also be formed as a result of replication errors or when DNA is damaged by oxidative stress [71].

## 3. DNA Repair Mechanisms

**Figure 5:**
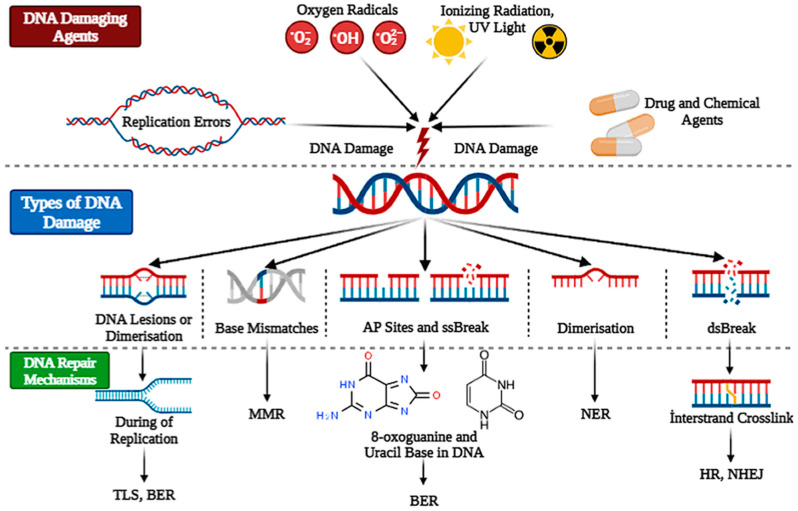
DNA damage and repair mechanisms. Various DNA-damaging agents cause a range of DNA lesions. Each is corrected by a specific DNA repair mechanism, namely mismatch repair, base-excision repair, translesion synthesis (TLS), transcription-coupled/global genome repair, or HR/NHEJ.

DNA repair mechanisms are processes by which cells detect and correct damage to the DNA molecules that encode their genomes. In human cells, both normal metabolic activities and environmental factors such as blue light and radiation can cause DNA damage, resulting in as many as one million individual molecular lesions per cell per day [72]. These lesions can include base modifications, sugar damage, and single- or double-strand breaks. DNA repair mechanisms are essential for the maintenance of genomic integrity, and their failure can lead to mutations, cell death, and cancer. There are several types of DNA repair mechanisms, including base excision repair (BER) nucleotide excision repair (NER), mismatch repair (MMR), homologous recombination (HR), and non-homologous end joining (NHEJ) (Figure 5). Each of these mechanisms has a specific role in repairing different types of DNA damage [73,74].

### 3.1. Base Excision Repair

This is the most common DNA repair mechanism and involves the removal of damaged bases from DNA strands. Base excision repair is a type of DNA repair process that occurs when a single base in the DNA strand is damaged. The damaged base is removed and replaced with a new base that is complementary to the original strand. This process is carried out by enzymes called DNA glycosylases, which recognize and remove the damaged base. After the damaged base is removed, the gap is filled in with the correct base by DNA polymerase, and the strand is sealed with DNA ligase [75]. This type of repair is important for maintaining the integrity of the DNA strands and preventing mutations.

#### 3.1.1. Structural Basis for Human Base Excision Repair

In one study, researchers aimed to provide insight into the structural basis for human BER by analyzing the crystal structures of DNA glycosylases and their complexes with DNA. The study reveals that the active sites of DNA glycosylases are highly conserved and that the glycosylases interact with DNA in a sequence-specific manner. The results of this study provide a better understanding of the molecular basis of BER and may help to develop new strategies for the treatment of genetic diseases caused by BER defects [76].

BER, which is triggered by DNA base damage, can be broken down into five main steps: (a) removing the damaged DNA base; (b) incising the subsequent abasic site; (c) tidying up the DNA ends; (d) putting the right nucleotide into the repair gap; and (e) ligating the last remaining nick in the DNA backbone [76,77]. Although it is believed that these stages are closely coordinated either through specific protein–protein interactions or through the development of repair complexes, each step is carried out by a distinct enzyme, or class of enzymes (discussed in the following section). The damaged base is excised in the first phase by a damage-specific DNA glycosylase by breaking of the N-glycosylic bond connecting the base to the sugar phosphate backbone. After that, AP endonuclease-1 (APE1) completes the second phase of BER by cutting the phosphodiester backbone 5′- to the abasic site to produce a DNA SSB with 3′-hydroxyl and 5′-deoxyribosephosphate (dRP) ends. The 8-oxoguanine DNA glycosylase (OGG1), which omits this second step by cleaving the DNA backbone to generate an SSB with variously changed DNA ends, is an example of a bifunctional DNA glycosylase that also has a mild lyase activity [78,79]. For instance, endonuclease VIII-like proteins (NEIL1-3) form a gap with 3′- and 5′ phosphate ends while OGG1 and endonuclease III-homologue (NTH1) create a gap with 3′- and 5′-unsaturated aldehyde and 5′-hydroxyl ends [80,81,82]. The DNA ends must be cleaned up in the third step of BER, which is carried out by enzymes (end processors) designed specifically for these ends. DNA polymerase β (Pol β) excises nicks with a 5′-dRP moiety produced by “traditional” BER, and APE1 and polynucleotide kinase phosphatase (PNKP). A new complementary nucleotide is added to the repair gap in the fourth phase of BER by Pol, and the remaining nick in the DNA backbone is then sealed in the last step by a complex of X-ray cross complementing protein-1 (XRCC1) and DNA ligase III [83,84,85,86].

#### 3.1.2. Role of the Base Excision Repair Pathway in Cancer

Several studies investigated the role of the BER pathway in cancer. The studies found that BER is important for maintaining genomic integrity and that defects in BER can lead to an increased risk of cancer. The studies also revealed that BER is important for the repair of DNA damage caused by chemotherapy and radiation, and that defects in BER can lead to an increased risk of chemotherapy and radiation resistance. The results suggest that BER may be a potential target for cancer therapy [87,88,89].

#### 3.1.3. Regulation of Base Excision Repair in Response to DNA Damage

Some studies investigated the regulation of BER in response to DNA damage. The studies find that BER is regulated by a variety of factors, including transcriptional and post-transcriptional regulation, as well as epigenetic modifications [90,91,92,93].

### 3.2. Nucleotide Excision Repair

Nucleotide excision repair (NER) is a type of DNA repair process that removes damaged or incorrectly paired nucleotides from a DNA strand. This repair process is important for maintaining the integrity of the genetic information stored in the DNA. NER is initiated when a DNA strand is damaged by ultraviolet (UV) radiation, chemical mutagens, or other environmental factors. The damaged region is identified and the DNA strand is cut at both ends of the damaged region. The damaged nucleotides are then removed and replaced with new nucleotides that match the original sequence. NER is a complex process that involves multiple proteins and enzymes. It is essential for maintaining the accuracy and stability of the genetic information stored in the DNA [94].

This repair process is essential for maintaining the integrity of the genome and preventing mutations that can lead to cancer [95,96]. NER is activated when DNA damage is detected, and it is able to remove a wide variety of lesions, including bulky adducts, UV-induced photoproducts, and oxidative damage. NER is also involved in the repair of DNA DSBs, which can be caused by ionizing radiation or certain chemotherapeutic agents. Mutations in genes involved in NER can lead to an increased risk of cancer. Defects in NER can result in an accumulation of DNA damage, which can lead to mutations and the development of cancer. For example, mutations in the XPD gene, which encodes a subunit of the NER complex, are associated with an increased risk of several types of cancer, including lung, bladder, and skin cancer [97,98,99]. In addition to its role in preventing cancer, NER is also important for the effectiveness of certain chemotherapeutic agents. Many chemotherapeutic agents cause DNA damage, and NER is responsible for repairing this damage. If NER is defective, the DNA damage caused by the chemotherapeutic agent may not be repaired, leading to an increased risk of cancer [100,101]. Therefore, it is important to ensure that NER is functioning properly in order to maximize the effectiveness of chemotherapy.

NER is a complex process that involves multiple proteins and enzymes. The regulation of NER in human cells is a complex process that is still not fully understood. The first step in NER is recognition of the damaged DNA. This step is performed by a group of proteins called the XPC-HR23B complex [102,103]. This complex recognizes the damaged DNA and recruits other proteins to the site of damage. These proteins include XPA, TFIIH, and XPG. Once the damaged DNA is recognized, the XPA protein binds to the damaged DNA and recruits other proteins to the site of damage. These proteins include XPG, XPB, XPD, and TFIIH. These proteins then form a complex called the transcription-coupled NER (TC-NER) complex. The TC-NER complex is responsible for removing the damaged DNA strand [104,105].

The next step in NER is the removal of the damaged DNA strand. This step is performed by the XPB and XPD proteins. These proteins unwind the DNA and create a gap in the DNA strand. The gap is then filled in by the XPG protein. Finally, the DNA is repaired by the ligation of the two strands of DNA, and this step is performed by the XPG protein. The ligation of the two strands of DNA results in the formation of a new DNA strand that is free of damage [103].

NER is essential for maintaining the integrity of the genome and preventing the accumulation of mutations. It is also important for protecting cells from the deleterious effects of aging [88]. Aging is associated with a gradual decline in the efficiency of NER, which can lead to an accumulation of DNA damage and mutations. This can lead to a variety of age-related diseases, such as cancer, neurodegenerative disorders, and cardiovascular diseases. NER is also involved in the aging process itself, as it is responsible for removing damaged proteins and other cellular components that accumulate over time. By maintaining the integrity of the genome, NER helps to protect cells from the deleterious effects of aging. As such, it is an important part of the aging process, and its role in maintaining health and longevity should not be overlooked [106].

In recent years, it has become increasingly clear that NER plays an important role in the development and progression of neurodegenerative diseases. For example, mutations in the genes involved in NER have been linked to a number of neurological disorders, including Huntington’s disease, spinocerebellar ataxia, and amyotrophic lateral sclerosis. In addition, NER has been shown to be impaired in patients with Alzheimer’s disease, Parkinson’s disease, and other neurodegenerative disorders [107]. The exact mechanism by which NER contributes to neurodegeneration is still unclear. However, it is thought that impaired NER leads to the accumulation of DNA damage, which can lead to the formation of toxic proteins and other cellular abnormalities. In addition, NER may be involved in the clearance of damaged proteins, which could prevent their accumulation and reduce the risk of neurodegeneration.

During NER, the damaged or incorrect nucleotides are identified and removed from the DNA strand by a complex of proteins known as the NER machinery. This machinery recognizes and binds to the damaged DNA, and then uses endonucleases to cut out the damaged nucleotides. The gap left behind is then filled in with the correct nucleotides, and the DNA strand is sealed back together. NER is important for DNA replication because it helps to ensure that the genetic information stored in the DNA is accurate and that the DNA is not damaged during the replication process [108,109]. Without NER, the genetic information stored in the DNA could be corrupted or lost, leading to mutations and other genetic diseases. NER also helps to prevent the accumulation of mutations over time, which can lead to cancer and other diseases.

### 3.3. Mismatch Repair

Mismatch repair (MMR) is a type of DNA repair process that corrects errors that occur during DNA replication. It works by identifying and correcting mismatches between the two strands of DNA. MMR is important for maintaining the integrity of the genome and preventing mutations that can lead to cancer and other diseases [110]. MMR is carried out by a complex of proteins, including MutS, MutL, and MutH. MutS recognizes the mismatched base pairs, MutL helps to separate the strands, and MutH cleaves the mismatched strand [111,112,113]. The correct nucleotide is then inserted into the gap and the strands are rejoined.

In normal cells, MMR genes help to ensure that the genetic information is accurately replicated and passed on to daughter cells. When MMR genes are mutated or not functioning properly, errors in DNA replication can occur, leading to genetic instability and an increased risk of cancer. Mutations in MMR genes are associated with a number of different types of cancer, including colorectal, prostate, lung, ovarian, and gastric cancer [114,115,116]. MMR genes are involved in the repair of mismatches in DNA strands that occur during DNA replication. When a mismatch occurs, MMR genes recognize the error and initiate a repair process. During this process, the mismatched nucleotides are removed and replaced with the correct nucleotides. This process helps to ensure that the genetic information is accurately replicated and passed on to daughter cells. In addition to their role in DNA repair, MMR genes also play an important role in regulating cell division. When MMR genes are functioning properly, they help to ensure that cells divide in a controlled and orderly manner. However, when MMR genes are mutated or not functioning properly, cells can divide in an uncontrolled manner, leading to the formation of tumors. Therefore, it is important to identify and address any mutations in MMR genes in order to reduce the risk of cancer [117].

Mismatch repair deficiency and microsatellite instability (MSI) are two important genetic features of colorectal cancer (CRC) [118]. MMR is a process that repairs errors in DNA replication, while MSI is a type of genetic instability caused by the inability of MMR to repair errors. MMR deficiency is associated with a higher risk of developing CRC, as well as other types of cancer [119]. MSI is also associated with a higher risk of developing CRC, and is often seen in tumors with MMR deficiency [120]. MSI is caused by the inability of MMR to repair errors in DNA replication. MSI is seen in tumors with MMR deficiency and is associated with a higher risk of developing CRC. MSI can also be caused by other factors, such as environmental exposures, certain medications, and certain lifestyle factors [121]. Both MMR deficiency and MSI are important genetic features of CRC, and both are associated with an increased risk of developing the disease. It is important to understand these genetic features in order to better diagnose and treat CRC.

Lynch syndrome is an inherited disorder caused by a mutation in one of the genes responsible for repairing errors in DNA replication. People with Lynch syndrome have an increased risk of developing colorectal, endometrial, ovarian, stomach, small intestine, and other types of cancer. People with Lynch syndrome may also have an increased risk of developing MMR-deficiency-related cancers [122,123].

Hereditary non-polyposis colorectal cancer (HNPCC) is a type of colorectal cancer caused by a mutation in one of the MMR genes. MMR is a process that helps to repair errors that occur during DNA replication. When a mutation occurs in one of the MMR genes, the body is unable to repair errors in DNA replication, leading to an increased risk of cancer. HNPCC is the most common type of colorectal cancer caused by a mutation in an MMR gene and is responsible for up to 3% of all colorectal cancer cases. People with HNPCC are at an increased risk of developing other types of cancer, including endometrial, ovarian, stomach, small intestine, and urinary tract cancers [124]. Treatment for HNPCC typically involves surgery, chemotherapy, and radiation therapy.

### 3.4. Non-Homologous End Joining

Non-homologous end joining (NHEJ) is a type of DNA repair pathway that repairs DSBs in DNA without the need for a homologous template. It is the major pathway for repairing DSBs in mammalian cells and is also found in other eukaryotes. NHEJ involves the direct ligation of the broken ends of the DNA molecule and is therefore a type of end-joining repair. NHEJ is important for maintaining the integrity of the genome, and defects in this pathway can lead to chromosomal instability and cancer [125].

#### 3.4.1. The Role of DNA-Dependent Protein Kinase Catalytic Subunit in NHEJ

DNA-dependent protein kinase catalytic subunit (DNA-PKcs) is a key enzyme involved in NHEJ. DNA-PKcs is a serine/threonine protein kinase that is activated by the binding of DNA ends to its Ku heterodimer. DNA-PKcs is essential for the recognition and binding of the DNA ends, as well as for the recruitment of other factors involved in NHEJ. DNA-PKcs also plays a role in the phosphorylation of the Ku heterodimer, which is necessary for the recruitment of the DNA-dependent protein kinase catalytic subunit (DNA-PKcs) to the DNA ends [126]. DNA-PKcs then phosphorylates and activates the DNA-dependent protein kinase catalytic subunit (DNA-PKcs), which is required for the assembly of the NHEJ complex. DNA-PKcs is also involved in the regulation of the NHEJ pathway by phosphorylating and activating the DNA-dependent protein kinase catalytic subunit (DNA-PKcs). DNA-PKcs is also involved in the regulation of the NHEJ pathway by phosphorylating and activating the DNA-dependent protein kinase catalytic subunit (DNA-PKcs). DNA-PKcs is also involved in the regulation of the NHEJ pathway by phosphorylating and activating the DNA-PKcs. Finally, DNA-PKcs is also involved in the regulation of the NHEJ pathway by phosphorylating and activating the DNA-PKcs [127]. Thus, DNA-PKcs plays a critical role in the NHEJ pathway by regulating the recruitment and activation of the DNA-PKcs.

#### 3.4.2. The Role of Ku70/80 in NHEJ

Ku70/80 is a heterodimeric protein complex that plays a key role in the repair of double-stranded DNA breaks by NHEJ [128]. This repair process is a major pathway for repairing DNA damage caused by ionizing radiation, reactive oxygen species, and other environmental agents. Ku70/80 binds to the DNA ends and recruits other proteins involved in the NHEJ process, including DNA-dependent protein kinase (DNA-PK), DNA ligase IV, and XRCC4 [129,130,131]. Ku70/80 also helps to stabilize the DNA ends and protect them from exonuclease digestion. In addition, Ku70/80 helps to facilitate the alignment of the two DNA ends prior to ligation [131]. Together, these activities of Ku70/80 are essential for the efficient and accurate repair of DNA DSBs by NHEJ.

#### 3.4.3. The Role of XRCC4 in NHEJ

XRCC4 is a protein that plays an important role in the NHEJ pathway of DNA repair. NHEJ is a major pathway used by cells to repair DSBs in DNA. XRCC4 is a scaffolding protein that helps to recruit other proteins to the site of the DSB. It also helps to stabilize the broken ends of the DNA and facilitates the ligation of the two DNA strands. XRCC4 is essential for the NHEJ pathway, and its absence leads to increased sensitivity to DNA-damaging agents and increased rates of chromosomal aberrations [131].

#### 3.4.4. The Role of DNA Ligase IV in NHEJ

DNA ligase IV is an enzyme that plays a critical role in the NHEJ pathway of DNA DSB repair. DNA ligase IV is responsible for joining the two ends of a DSB together by forming a phosphodiester bond. It does this by recognizing and binding to the ends of the DSB, and then catalyzing the formation of a phosphodiester bond between the two ends [130,132]. This process is essential for the repair of DSBs, as it allows for the restoration of the original sequence of the DNA molecule.

#### 3.4.5. The Role of Artemis in NHEJ

Artemis is a DNA repair enzyme that plays an important role in NHEJ. NHEJ is a major pathway for repairing double-strand breaks in DNA. Artemis is a key component of the NHEJ pathway, as it is responsible for recognizing and processing the broken ends of the DNA. It then helps to ligate the two ends together, forming a new DNA strand. Without Artemis, NHEJ would be unable to repair double-strand breaks, leading to cell death or mutations [133].

#### 3.4.6. The Role of DNA Polymerase in NHEJ

DNA polymerase is an essential enzyme in the process of NHEJ. NHEJ is a type of DNA repair mechanism that repairs double-stranded breaks in DNA without the need for a homologous template. During NHEJ, DNA polymerase is responsible for filling in the gaps created by the double-stranded break. DNA polymerase is also responsible for proofreading the newly synthesized DNA strands to ensure that the repair is accurate. Finally, DNA polymerase is also involved in ligation of the newly repaired DNA strands [134].

#### 3.4.7. The Role of Microhomology in NHEJ

Microhomology, which is defined as short stretches of nucleotide sequence similarity between the two ends of a DSB, has been shown to play an important role in NHEJ. Microhomology can enhance the efficiency of NHEJ by providing a short stretch of sequence complementarity that can be used to align the two ends of the DSB. This alignment facilitates the formation of a stable DNA duplex, which increases the efficiency of ligation. Microhomology can also increase the accuracy of NHEJ by providing a short stretch of sequence complementarity that can be used to distinguish between the two ends of the DSB, thus preventing the formation of incorrect ligation products. Finally, microhomology can also increase the fidelity of NHEJ by providing a short stretch of sequence complementarity that can be used to ensure that the correct ligation products are formed [135,136].

#### 3.4.8. The Role of Chromatin Structure in NHEJ

Chromatin structure plays an important role in NHEJ, as it can affect the accessibility of the DNA ends to the NHEJ machinery. Chromatin structure can also affect the efficiency of NHEJ, as it can influence the ability of the NHEJ machinery to recognize and join the DNA ends. Chromatin structure can also affect the fidelity of NHEJ, as it can influence the ability of the NHEJ machinery to distinguish between correct and incorrect DNA ends. Finally, chromatin structure can also affect the speed of NHEJ, as it can influence the ability of the NHEJ machinery to locate and join the DNA ends [137,138].

### 3.5. Homologous Recombination

Homologous recombination (HR) DNA repair is a type of DNA repair process that occurs when a double-stranded break in DNA occurs. This type of repair utilizes homologous sequences from a sister chromatid or a homologous chromosome to repair the broken DNA strand. During this process, the broken ends of the DNA are resected, and then the homologous sequences are used as a template to repair the broken strands. The repaired DNA is then ligated back together to form a new, intact DNA molecule [139]. Homologous recombination DNA repair is an important process for maintaining the integrity of the genome and preventing mutations.

#### 3.5.1. The Role of Homologous Recombination in DNA Repair and Genome Stability

HR is an important process in DNA repair and genome stability. It is a type of genetic recombination in which nucleotide sequences are exchanged between two similar or identical molecules of DNA. HR is used to repair double-stranded breaks in DNA, which can occur due to environmental damage, such as UV radiation or chemical mutagens. It is also used to replace a damaged or mutated gene with a healthy copy, which helps to maintain the stability of the genome. HR is also important for the maintenance of genetic diversity and the evolution of species. It is used to shuffle genetic material between chromosomes and between different species, allowing for the creation of new gene combinations. HR is essential for the maintenance of genome stability and the repair of DNA damage, and is thus an important process in the preservation of life [140,141,142,143].

#### 3.5.2. Homologous Recombination-Mediated DNA Repair Pathways in Bacteria

HR-mediated DNA repair pathways are essential for the maintenance of bacterial genome integrity. Without these pathways, bacteria would be unable to repair DSBs in DNA, correct errors in DNA replication, and recombine DNA fragments during transformation and conjugation. Homologous recombination is a process by which DNA strands are exchanged between two homologous DNA molecules. This process is used by bacteria to repair DSBs in DNA, as well as to correct errors in DNA replication. Homologous recombination is also used to recombine DNA fragments during bacterial transformation and conjugation [144].

The HR-mediated DNA repair pathways in bacteria involve several proteins, including RecA, RecBCD, and RuvABC [145,146,147]. RecA is a DNA-binding protein that is responsible for the pairing of homologous DNA strands. RecBCD is a complex of three proteins that is responsible for unwinding and processing DNA strands during recombination. RuvABC is a complex of three proteins that is responsible for resolving Holliday junctions, which are intermediates in homologous recombination. In addition to these proteins, bacteria also contain a variety of other proteins that are involved in HR-mediated DNA repair pathways. These proteins include DNA polymerases, DNA ligases, and exonucleases [148]. DNA polymerases are responsible for replicating DNA strands during recombination. DNA ligases are responsible for joining the newly replicated DNA strands. Exonucleases are responsible for removing nucleotides from the ends of DNA strands.

#### 3.5.3. Homologous Recombination-Mediated DNA Repair in Eukaryotes

HR-mediated DNA repair occurs through the exchange of genetic material between two homologous DNA strands. This exchange of genetic material is facilitated by a group of proteins known as recombinases. These proteins recognize specific sequences of DNA and catalyze the exchange of genetic material between the two strands. The process of HR-mediated DNA repair is essential for the survival of eukaryotic organisms, as it helps to maintain the integrity of the genome and prevent the accumulation of genetic mutations [149].

#### 3.5.4. The Role of Homologous Recombination in Cancer Development

HR has been implicated in the development of cancer in several ways [150]. First, HR is involved in the repair of DNA DSBs, which can occur due to exposure to environmental agents such as radiation or chemicals. If these breaks are not repaired correctly, they can lead to mutations that can contribute to the development of cancer. Second, HR can also contribute to the development of cancer by allowing for the exchange of genetic material between two different chromosomes. This can lead to the formation of translocations, which are chromosomal rearrangements that can result in the activation of oncogenes or the inactivation of tumor suppressor genes [151]. These changes can lead to the development of cancer. Finally, HR can also contribute to the development of cancer by allowing for the generation of genetic diversity during meiosis [152]. This can lead to the formation of aneuploidy, which is an abnormal number of chromosomes, which can result in the activation of oncogenes or the inactivation of tumor suppressor genes.

### 3.6. Translesion Synthesis

Translesion synthesis (TLS) is a type of DNA repair that occurs when a DNA polymerase is blocked by a lesion in the DNA template strand [153]. The blocked polymerase is unable to replicate the template strand, so a specialized polymerase, called a translesion polymerase, is recruited to bypass the lesion and complete the replication of the template strand. This process is important for maintaining the integrity of the genome, as it allows for the replication of damaged DNA. Translesion synthesis is a last resort, as it is error-prone and can lead to mutations [154].

#### 3.6.1. Structural Basis of Translesion Synthesis by DNA Polymerase H

DNA polymerase η is a specialized DNA polymerase that is responsible for TLS. The structural basis of TLS by DNA polymerase η has been studied extensively and is thought to involve a number of different steps [155,156,157]. First, the enzyme binds to the damaged DNA and forms a ternary complex with the DNA and a dNTP. This allows the enzyme to scan the DNA and identify the site of damage. Once the damage is identified, the enzyme can then make the necessary adjustments to the active site to accommodate the damaged base. This includes adjusting the position of the active site residues and forming hydrogen bonds with the damaged base. Once the active site is properly adjusted, the enzyme can then catalyze the formation of a phosphodiester bond between the incoming dNTP and the damaged base. This is followed by the formation of a second phosphodiester bond between the incoming dNTP and the next undamaged base. This allows the enzyme to bypass the damaged base and continue DNA replication. Finally, the enzyme can then catalyze the formation of a third phosphodiester bond between the incoming dNTP and the next undamaged base. This allows the enzyme to complete the process of TLS and continue DNA replication [156]. Overall, the structural basis of TLS by DNA polymerase η involves a number of different steps that allow the enzyme to identify and bypass a site of DNA damage. This allows the cell to survive and repair the damage, allowing for the continued replication of the DNA.

#### 3.6.2. Translesion Synthesis and the Role of DNA Polymerase H in Cancer

Studies have shown that Pol η is overexpressed in many types of cancer, suggesting that it plays an important role in tumorigenesis [158,159]. Pol η is able to bypass DNA damage by inserting a single nucleotide opposite a damaged base. This process is known as TLS and is important for maintaining genomic integrity. Pol η is able to bypass a wide range of DNA lesions, including those induced by UV radiation, alkylating agents, and oxidative damage. This ability to bypass DNA damage makes Pol η an important enzyme for maintaining genomic integrity and preventing mutations that can lead to cancer. Studies have shown that Pol η is able to bypass DNA damage more efficiently than other polymerases [160], allowing it to facilitate the accumulation of mutations that can lead to cancer. In addition, Pol η is able to bypass DNA damage more efficiently in the presence of certain cancer-promoting proteins [161], suggesting that it may be involved in the development of cancer. Overall, Pol η is an important enzyme for maintaining genomic integrity and preventing mutations that can lead to cancer. Its overexpression in many types of cancer suggests that it plays an important role in tumorigenesis, likely by facilitating the accumulation of mutations that can lead to cancer.

#### 3.6.3. Translesion Synthesis and Its Role in the Development of Antibiotic Resistance

TLS is important in the development of antibiotic resistance in bacteria, as it allows the bacteria to survive exposure to antibiotics that would otherwise kill them [162]. TLS is important in the development of antibiotic resistance in bacteria, as it allows the bacteria to survive exposure to antibiotics that would otherwise kill them. In the presence of an antibiotic, the DNA polymerase enzyme is able to recognize the damage to the DNA template and switch to a TLS-capable polymerase. This polymerase is able to bypass the damage and replicate the DNA, allowing the bacteria to survive the antibiotic. The development of antibiotic resistance is a major concern in the medical community, as it can lead to the emergence of “superbugs” that are resistant to all known antibiotics. TLS is one of the main mechanisms by which bacteria can develop antibiotic resistance, and understanding this process is essential for developing strategies to combat antibiotic resistance [163].

#### 3.6.4. Translesion Synthesis and Its Role in the Development of Viral Pathogenesis

TLS is important for the survival of viruses, as it helps them to overcome the effects of DNA damage caused by environmental factors such as UV radiation, oxidative stress, and mutagenic chemicals [164]. TLS helps to increase the mutation rate of the virus, which can lead to the emergence of new viral variants that are more resistant to antiviral drugs. TLS is also important for the development of viral pathogenesis, as it allows viruses to replicate and spread despite the presence of DNA damage [165]. This is especially important for viruses that cause chronic infections, as they require a high level of genetic diversity to survive and spread. TLS also helps to increase the mutation rate of the virus, which can lead to the emergence of new viral variants that are more resistant to antiviral drugs.

## 4. Pathophysiological and Therapeutic Implications of Oxidative DNA Damage and Repair

Oxidative DNA damage (and especially 8-oxoG/8-oxodG) is more than a passive marker of cellular stress—it directly dictates disease trajectories by altering mutation spectra, modulating transcriptional programs, and engaging inflammatory signaling cascades. Elevated 8-oxodG is associated with tumor burden, neurodegeneration, and metabolic disease in multiple clinical cohorts, and is used routinely as a non-invasive biomarker in urine and plasma assays. Recent reviews consolidate 8-oxodG’s role as a diagnostic/prognostic marker and readout for therapeutic response in redox-targeted therapies [34].

Mechanistically, the interface between oxidized guanine lesions and repair enzymes has been identified as a pathogenic nexus. OGG1 has dual function: in addition to canonical BER activity, it can also act as an epigenetic reader and control NF-κB and other inflammatory pathways. This “molecular duality” explains why OGG1 activity can be protective in some contexts (preventing mutagenesis) but pro-pathogenic in others (driving chronic inflammation and transcriptional programs that promote tumorigenesis and vascular dysfunction). Inhibiting OGG1 therefore presents a nuanced challenge: selective inhibition can suppress pro-inflammatory signaling and block tumor cell proliferation in defined genetic/physiological contexts, while the loss of repair function must be modulated so as not to unleash unwanted mutagenesis [166].

Aging is tightly linked to oxidative DNA damage by cumulative lesion load, telomere shortening, and diminishing repair capacity. Cross-sectional and longitudinal investigations demonstrate elevated levels of 8-oxo lesions and oxidized RNA species in aging tissues and also in diseases of aging (e.g., neurodegeneration, age-related macular degeneration), showing that imperfect removal of oxidative lesions contributes to functional decline [167]. These findings support the view that modulation of redox balance and modulation of repair capacity are viable strategies to slow age-related pathologies, but must be precisely calibrated to avoid the promotion of genomic instability.

Recent evidence suggests that DNA repair machineries are closely related to longevity-regulating networks such as SIRT1, PARP1, and FOXO transcription factors [168]. SIRT1 deacetylates certain BER enzymes and activates repair, while PARP1 consumes NAD^+^ during single-strand break repair [169]. Excessive activation of PARP1 in response to prolonged oxidative stress depletes cellular pools of NAD^+^, impairing mitochondrial function and causing age-related metabolic dysfunction. Longevity interventions, including caloric restriction, resveratrol, and NAD^+^ precursors (e.g., nicotinamide riboside), increase DNA repair efficiency through SIRT–PARP signaling modulation and mitochondrial biogenesis, emphasizing the repair–metabolism axis for healthy aging (Table 1) [170]. Age-associated oxidative accumulation of DNA lesions is causally related to age-related diseases like cancer, Alzheimer’s disease, Parkinson’s disease, and atherosclerosis. Oxidative DNA and RNA modifications in neurodegenerative diseases interfere with transcriptional integrity and synaptic function. Defects in repair enzymes such as OGG1 and NEIL1 have been linked with cognitive decline and increased neuronal apoptosis [171]. In the cardiovascular system, oxidative DNA damage in endothelial cells increases vascular dysfunction and inflammation, accelerating atherogenesis [172]. These observations verify the concept that preservation of repair capacity is not only crucial for the prevention of cancer but also for the preservation of neuronal and vascular integrity with aging. Therapeutic approaches that modulate redox homeostasis and DNA repair are also beginning to emerge as new approaches for combating age-related illnesses.

Low-molecular-weight compounds that stimulate BER or trigger antioxidant protection, including mitochondrial-targeted antioxidants (MitoQ, SkQ1) or SIRT1 inducers, can slow oxidative damage accumulation [173]. Furthermore, PARP inhibitors originally intended to be used against BRCA-mutated cancers are being investigated for their anti-aging effects to determine how long-term modulation of DDR affects aging and metabolic health. With recent breakthroughs in omics-based lesion mapping and CRISPR models, age-related repair dynamics can now be analyzed at single-cell and tissue levels to gain mechanistic insights into how DNA repair decline guides aging trajectories.

## 5. Targeting DNA Damage Response and Oxidative DNA Repair Pathways in Cancer Therapy

Cancer cells experience genotoxic, constitutive oxidative stress due to increased metabolic activity, mitochondrial dysfunction, and oncogene-driven signaling, resulting in aberrant ROS generation and DNA damage. In order to survive under these conditions of genotoxic stress, tumor cells strongly rely on efficient DNA repair networks, particularly BER and the DDR signaling pathways to maintain viability and resist therapy-induced cytotoxicity. This reliance creates a therapeutic window: selective inhibition of repair mechanisms can sensitize tumor cells to oxidative and chemotherapeutic stress without damaging normal cells [174,175].

BER pathway plays a vital role in maintaining the viability of cancer cells and promoting therapeutic resistance [174]. Inhibition of BER has been one of the promising anticancer strategies, as its inhibition inhibits tumor cells’ ability to repair chemoradiotherapy-caused DNA damage [176]. Conventional BER inhibitors primarily target such essential enzymes as poly (ADP-ribose) polymerase 1 (PARP1), APE1, and DNA polymerase β (Polβ), thereby enhancing the cytotoxic effect of genotoxic therapy and helping evade acquired resistance. PARP inhibitors, for example, have demonstrated noteworthy clinical advantage when used to treat BRCA-mutated breast and ovarian cancer based on the synthetic lethality principle. The recent development in targeted therapies has witnessed new approaches such as proteolysis-targeting chimeras (PROTACs) and photodynamic therapy (PDT) involving photosensitizers. PROTACs enable selective protein degradation of BER-related proteins, which ensures long-term and full-range inhibition compared to conventional small-molecule inhibitors [177]. This new approach vows to overcome adaptive resistance mechanisms [178]. Moreover, PDT has also drawn interest as a result of its capability for spatial control of the activation of photosensitizers in conjunction with selective inhibition of BER. Following activation by specific wavelengths of light, such photosensitizers generate ROS and hence induce oxidative DNA damage in addition to interfering with BER-mediated repair mechanisms, thereby increasing overall anticancer efficacy at less systemic toxicity [179].

Single-strand selective monofunctional uracil-DNA glycosylase 1 (SMUG1) initiates BER by excising uracil residues from single-strand DNA, including U:G mismatches and a number of oxidized pyrimidine derivatives [180]. Experimental evidence indicates that SMUG1 and uracil-DNA glycosylase (UNG) act synergistically in uracil excision in vivo, emphasizing the central role of SMUG1 in ensuring genomic stability [181]. Moreover, the fact that UNG/SMUG1 double knockout mice show cytosine-to-thymine (C → T) transitions demonstrates that SMUG1 contributes not only to antimutagenic defense but also to regulation of transcription. Growing evidence demonstrates that SMUG1 expression influences cancer susceptibility as well as response to treatment. SMUG1 is frequently overexpressed in breast cancer tissues and cancer cell lines. Immunohistochemical analysis of breast cancer tissue arrays revealed that low SMUG1 expression correlates with highly aggressive tumor phenotypes, for which it has been suggested as an independent prognostic biomarker in ER-positive breast cancer and a predictive marker of response to adjuvant chemotherapy [181].

Khanna proposed that distinct thresholds for DNA damage exist at various stages of tumorigenesis and stressed the central role of the DDR pathway in human cancers [182]. The DDR is a rigidly controlled and well-coordinated system that rapidly responds to DNA damage in normal and cancer cells, and therefore implies that some DDR components may represent valid targets for therapeutic intervention against cancer cell proliferation. A striking feature of cancer cells is that the individual components of DDR, but not in normal tissue, are deficient, thereby leaving the remaining compensatory pathways fully dependent on the tumor cells. These backup DDR pathways enable cancer cells to survive in the elevated ROS and replicative stress of the tumor microenvironment. Hence, therapeutic inhibition of these backup DDR pathways can selectively sensitize cancer cells to DNA-damaging agents, which enhances cytotoxicity while sparing normal tissue. Inhibition of DDR is therefore a promising approach for both augmenting existing therapies and sensitizing resistance to conventional therapies [183,184,185,186].

Several very selective inhibitors of DNA repair pathways are now at advanced stages of preclinical and clinical development. Of these, poly (ADP-ribose) polymerase inhibitors (PARPis) represent the first clinically proven class of DDR-targeted drugs to be engineered to take advantage of synthetic lethality [187,188]. In recent research, oxidative stress has been found to induce DNA strand breaks and PARP-1 activation, leading to mitochondrial ROS production and cell death. PARPis, however, block ROS-mediated apoptosis by preserving mitochondrial membrane potential via ATF4/MKP-1–mediated inhibition of JNK and p38 MAP kinases processes associated with cancer stem cell maintenance. This activity may be behind the clinical efficacy of PARPis in combination with ROS-modulating drugs [189]. DDR-targeting agents, such as PARPis, have proven to be able to enhance platinum agent-induced DNA damage and replication stress to enhance treatment effectiveness in advanced cancers [190]. Further studies of cell-cycle checkpoint signaling, particularly the ataxia–telangiectasia mutated (ATM) and ataxia–telangiectasia and Rad3-related (ATR) pathways, have created very potent and selective inhibitors already in preclinical and clinical development. ATR inhibitors (ATRis), for example, exhibited synthetic lethality in DDR-deficient cancer cells and are under investigation as monotherapies and in combination with platinum agents, PARPis, and checkpoint blockade immunotherapy [184,186]. Preclinical studies have also identified wild-type p53-induced phosphatase 1 (WIP1; also known as protein phosphatase 2C delta) as a putative DDR target. WIP1, p53-inducible in response to genotoxic stress, is a negative p53 pathway regulator through the removal of DDR proteins such as p53 and γH2AX phosphorylation. Overexpression of WIP1 inhibits p53-mediated tumor suppression and cooperates with oncogenes to facilitate tumorigenesis, whereas loss of WIP1 inhibits tumor growth and reactivates p53-mediated cell cycle control. Pharmacologic inhibition of WIP1 with the small molecule compound GSK2830371 rescues p53 signaling and triggers cell death or senescence in cancer cells, but not in normal cells with low basal levels of WIP1. Furthermore, the combination of WIP1 inhibition with DNA-damaging chemotherapy or MDM2 inhibitors (e.g., nutlin-3) generated synergistic cytotoxicity in several tumor models [191].

## 6. Emerging Technologies: CRISPR-Based Tools and Omics Approaches

Recent advances in CRISPR-mediated genome editing have greatly facilitated mechanistic studies of DDR. The CRISPR/Cas9 system enables the induction of precise DSBs or single-base damage at targeted genomic locations, thereby allowing direct visualization of repair protein recruitment and kinetics to damage sites [192,193]. Engineered derivatives such as Cas12a (Cpf1) and base editors further expand the possibility to model different types of DNA lesions and study their repair pathways [194,195]. CRISPR interference (CRISPRi) and activation (CRISPRa) systems are also used to perturb DDR genes in a systematic manner, uncovering novel regulators involved in NHEJ, HR, and MMR [196,197].

Recent advances in omics technologies have greatly enhanced our understanding of DNA damage and repair mechanisms. Genomic tools, particularly next-generation sequencing (NGS), allow one to detect DNA lesions at single-nucleotide resolution and mutational signatures genome-wide. The key genes involved in DDR include ATM, ATR, BRCA1, and TP53, which code for proteins that serve as sensors, transducers, and effectors for repair mechanisms [198]. For example, ATM and ATR kinases phosphorylate their downstream targets like CHK1 and CHK2, which regulate cell cycle checkpoints and repair pathways. RNA-seq transcriptome studies report on dynamic gene expression regulation upon DNA damage. Transcriptome studies using RNA-seq have revealed how cells control transcription of repair gene transcripts like RAD50 and MRE11 during genotoxic stress [199]. Proteomics, as in mass spectrometry-based studies, enables the quantification and identification of post-translational modifications to DNA repair proteins. Phosphorylation, ubiquitination, and SUMOylation are notable modifications that regulate protein stability, localization, and function in the context of the DDR [200]. For instance, ubiquitination by the E3 ligase MDM2 regulates p53 stabilization to act as a transcription factor for cell cycle arrest and apoptosis. Integrative omics approaches, which incorporate genomics, transcriptomics, and proteomics, have also revealed the complexity of DNA repair networks and the context-dependent activation in different cell types or stress conditions [198]. The approaches also provide the means for identifying disease susceptibility biomarkers, therapeutic targets, and impacts of environmental and endogenous factors on genome integrity. Structural proteomics and imaging techniques also provide spatial and temporal details on repair complex assembly at DNA damage sites.

## 7. Conclusions and Perspectives

DNA integrity is continuously targeted by oxidative stress and environmental genotoxins. Efficient DNA repair processes such as BER, NER, and MMR are crucial for genomic stability and disease prevention. Dysregulation or breakdown of these repair processes causes cancer initiation and promotion, neurodegenerative, and cardiovascular disease. Elucidation of molecular mechanisms of oxidative DNA damage repair offers pivotal information in risk assessment, prevention, and the development of targeted therapies. New technologies such as CRISPR genome editing, single-cell genomics, and integrative omics provide unprecedented capabilities to map DNA repair dynamics and reveal new biomarkers of disease susceptibility. Future directions should involve translating the mechanistic understanding into clinical applications, including predictive diagnosis and personalized medicine leveraging DNA repair modulation for disease prevention and treatment.

## Figures and Tables

**Table 1:** Oxidative DNA Lesions, Repair Pathways, and Associated Human Diseases.

Lesion Type	Primary Repair Pathway	Key Enzymes	Associated Diseases/Biological Consequences
8-oxoGuanine (8-oxoG)	BER	OGG1, MUTYH, APE1	MUTYH-associated polyposis, colorectal cancer, carcinogenesis
Oxidation of thymine to 5-hydroxyuracil (5-hU)	BER/NER (minor contribution)	NTHL1, NEIL1, APE1	Aging-related genomic instability, neurodegeneration
AP site (apurinic/apyrimidinic site)	BER	APE1, XRCC1, DNA polymerase β	Genomic instability, increased mutation frequency
Single-strand break (ssBreak)	SSBR/PARP-mediated repair	PARP1, XRCC1, DNA ligase III	Ataxia, neurodegenerative disorders
